# Mental Work as a Young Man's Career

**Published:** 1912-09-07

**Authors:** 


### MENTAL WORK AS A YOUNG MAN'S CAREER.

### The Life and Progress of the Assistant Medical Officer.

At the present time much more attention is being given in our medical schools to the study of psychology and the treatment of mental diseases than was formerly the case. Now that a special degree in psychological medicine is being instituted, the attention of the student entering upon his career will be more directed to this particular branch of his profession. In the past it has been too much neglected. Every medical man should have some practical acquaintance with the manifestations of mental disease and its treatment. If this were so, many cases which medical men now hesitate to certify until they have reached a stage at which treatment is ineffective would be placed earlier under treatment, and the prospects of the recovery of the patient thereby increased. Some of the medical certificates under which patients are sent to mental hospitals show that there are medical men who are unable to express in a clear and concise form the evidence upon which they have formed their opinion. We have known a medical certificate to be given in which the chief ground for believing the patient to be insane was said to be that "she states that the blood of Jesus cleanses from all sin." This apparently had satisfied the certifier. About the insanity of the patient there could not be a shadow of a doubt, and a very short interview with her made it plain that the defect lay not in the judgment of the certifier but in his capability to express in plain language the grounds upon which he had formed it.

### Clinical Clerks and their Value.

It is to be hoped that the system of clinical clerks will be more extensively adopted in mental hospitals. We feel sure that it would be alike advantageous to those who held the posts and to the institutions in which they worked. There is so much routine to be gone through and so much wearisome clerical work to be performed by an assistant medical officer that he is an exceptional man indeed whose enthusiasm for medicine does not in time become somewhat damped. There is a natural tendency to give undue importance to the clerical part of his duties to the detriment of the more purely medical side of them. Here the clinical clerk might, to his own great benefit, be utilised. Unfortunately, good honest clinical work in our mental hospitals is not, to speak commercially, a paying line. It does not give so many opportunities for attracting attention to the worker, and thereby smoothing the pathway which leads to that superintendentship which is the goal of every assistant medical officer's ambition.

For men with a liking for pathology and its practical study this specialty should possess peculiar attractions. There is an abundance of material and already at some institutions there are laboratories in which every facility is given for special research and the student is encouraged to pursue original work. It is likely also that in the near future the establishment of joint laboratories by neighbouring authorities will increase these opportunities for good work in this line. A knowledge of the methods of bacteriology and some acquaintance with bio-chemistry must be included in the equipment of the ambitious candidate.

### The Pros and Cons of Mental Work.

There is much to be said for and against a man entering the mental hospital service. It has the great advantage of being a public service with provision for superannuation allowance. This, of course, must be borne in mind when comparing it financially with other branches of the profession, though we fear that the value of this provision for advancing years is not estimated fully by men at the age at which the career is usually entered upon. As a rule, the best men in the specialty come to the front, though it must be admitted that there is perhaps a larger element of luck in it than in some other fields of medical work. Perhaps luck plays a part less frequently than it appears to do, and that name is applied to the foresight shown by the beginner in his selection of the institution in which to make his debut.

The life of a mental hospital assistant medical officer, especially in a large institution, is a pleasant enough one for a year or two. Many medical men might with advantage devote that time to it. If, however, a man remain in it for some years and promotion be delayed, while no opportunity is given for marriage, a time comes when hope deferred maketh the heart very sick. Then the weary years of waiting begin to tell. There seems to be no hope of promotion in the service the assistant medical officer has taken up, and the years spent in it have certainly not increased his fitness for another branch of the profession. Medical superintendents, as well as assistant medical officers, are but human, and the former may feel and show that the institution under his charge would be, in his opinion, benefited by a change in the latter. Relations between them become strained, and working together in a limited area with many opportunities for friction the position may become almost intolerable. Many men will admit that this is not expressing the painfulness of the situation too strongly. It is hard to keep up a living interest in the work and to discharge loyally his duty to his chief and the institution in which he works when the assistant medical officer feels that the future is a blank before him.

### Financial Prospects and Marriage Facilities.

From a monetary point of view the service cannot be considered altogether unsatisfactory, for a few years at least. A career which offers a junior assistant medical officer £150 yearly, with a prospect of rising to £200; and to a senior assistant £200 rising to £250, with board and lodging, does not compare unfavourably with other openings for beginners. It is, however, when a man desires to make a home-life for himself, and begins to recognise there is little prospect of promotion, that the harness begins to gall its wearer. There are many assistants, and when considering the number of the superintendentships which fall vacant annually, it may truthfully be said, "What are they among so many?" A man, after five years' service, should take stock of his position and reckon up his prospects of promotion. The decision which lies before him is a momentous one, and probably has not its parallel in any other branch of the profession. It is imperative that the posts of senior assistant medical officer should be made of such value as will attract and retain good men in the service while giving facilities for marriage. By this means alone can the growing discontent among assistant medical officers at the slowness of promotion and the conditions of their life be allayed. After the first year or two the life of an asylum assistant medical officer is, paradoxical as it may seem, monotonous and trying. He is usually placed some distance from a town, and is thrown largely on his own resources. That there should be a constant flow of young medical men through our mental hospitals is necessary, and from every point of view, except that of the discipline in them, and that is an important exception, much to be desired. It is well that up-to-date ideas and methods should be brought before the senior men, and that they should thus be taught that medicine has moved since they were students. Their time is so taken up with administrative and routine duties that they have little opportunity of keeping abreast with the progress of their profession. Indeed, we feel strongly that so important is this aspect of the question that we would advocate a system of allowing senior men after every five years' service a period of leave, which should be spent in attending a course of study in some medical school.

The Successful Superintendent.

In addition to the purely medical work, much administrative capacity is demanded in a successful medical superintendent. This requires qualities often different from those which are requisite for a successful physician, and though there have been exceptions, yet it is surprising how often they have been found combined. This experience can only be gained by work in a mental hospital, and he is the wise superintendent who endeavours so far as possible to give his colleagues every opportunity of acquiring a knowledge of this branch of their work. A superintendent's life tends to produce an autocratic and self-sufficient frame of mind, the growth of which may be retarded by a judicious use of consultation with his junior colleagues. A sufficient number of questions which he alone can deal with and decide upon will still be left for him. It is not every man to whom this work is suitable. Anyone with a neurotic inheritance and upon whom the sense of responsibility weighs heavily would be well advised to shun it. Some outside interests are essential which will take him away from his everyday work. Without this the superintendent who is absorbed in his duty may lose the proper focus at which his responsibilities, great as they are, should be looked.

